# Differences in Verbal and Visuospatial Forward and Backward Order Recall: A Review of the Literature

**DOI:** 10.3389/fpsyg.2017.00663

**Published:** 2017-05-04

**Authors:** Enrica Donolato, David Giofrè, Irene C. Mammarella

**Affiliations:** ^1^Department of Developmental and Social Psychology, University of PadovaPadova, Italy; ^2^Department of Natural Sciences and Psychology, Liverpool John Moores UniversityLiverpool, UK

**Keywords:** order recall, verbal working memory, visuospatial working memory, short-term memory, neural correlates

## Abstract

How sequential, verbal and visuospatial stimuli are encoded and stored in memory is not clear in cognitive psychology. Studies with order recall tasks, such as the digit, and Corsi span, indicate that order of presentation is a crucial element for verbal memory, but not for visuospatial memory. This seems to be due to the different effects of forward and backward recall in verbal and visuospatial tasks. In verbal span tasks, performance is worse when recalling things in backward sequence rather than the original forward sequence. In contrast, when it comes to visuospatial tasks, performance is not always worse for a modified backward sequence. However, worse performance in backward visuospatial recall is evident in individuals with weak visuospatial abilities; such individuals perform worse in the backward version of visuospatial tasks than in the forward version. The main aim of the present review is to summarize findings on order recall in verbal and visuospatial materials by considering both cognitive and neural correlates. The results of this review will be considered in the light of the current models of WM, and will be used to make recommendations for future studies.

## Introduction

The ability to process serially ordered information is fundamental to many aspects of our lives, including spelling and orientation to a new environment. However, the cognitive mechanisms underlying encoding and recall of verbal and visuospatial sequences are still not fully understood.

One of the processes involved in serial recall is short-term memory (STM), which allows individuals to hold a small amount of information for a short period of time. Verbal STM is generally tested with the digit span task (DST) that involves recalling sequences of digits, while the ability to retrieve visuospatial information is typically tested with the Corsi span task (CST) that involves recalling sequences of blocks (Berch et al., 1998). In both verbal and visuospatial span tasks, participants may be asked to recall the information in either forward or backward order. In the DST, performance is usually worse in the backward version of the task (Baddeley, 1986; Li and Lewandowsky, 1995), while recall of the forward and backward versions of the CST is much the same for most subjects (Wilde and Strauss, 2002; Cornoldi and Mammarella, 2008). Although, these results give the impression that forward and backward verbal and visuospatial span tasks likely measure different constructs, experimental and neural correlated findings regarding serial recall tasks are not consistent, suggesting a need for further research. It is also important to carefully review the research and literature on this subject to-date. Hence, here we will summarize the findings from studies of forward and backward recall in verbal and visuospatial domains as it relates to both cognitive and neural correlates.

### Selection of studies

We conducted a literature search via PsychINFO, Web of Science and Google Scholar electronic databases. We used the following search keywords: serial/order recall, forward/backward span/recall, DST and CST, verbal/visuospatial, STM/WM. We searched for these terms in titles, in abstracts, and in the keyword lists themselves. Titles and abstracts were screened for appropriateness and independently reviewed for relevance. Papers published from January 1960 to September 2016 have been considered. 132 manuscripts were originally selected for scrutiny; ultimately, only 54 met our inclusion criteria and were considered in the present review.

Papers were considered for inclusion if they covered: (i) behavioral or neural correlates for forward and/or backward recall in the verbal and/or visuospatial domain; and (ii) the impact of verbal and/or visuospatial memory capacity. Studies focusing purely on theoretical models for memory systems were not considered.

### Similarities and differences in order recall in verbal or visuospatial domains

Studies in order recall on verbal and visuospatial domains to-date have used different methodologies and had different aims.

Forward and backward serial position curves have been analyzed by considering primacy and recency effects in verbal WM (Table 1, verbal WM). As for backward recall, findings have shown a qualitative change in serial position curves characterized by an increased recency effect and a decreased primacy effect (Li and Lewandowsky, 1993, 1995; Hulme et al., 1997, see also Penney, 1989 for a review). Likewise, in forward and backward visuospatial tasks, both primacy and recency effects also occur (Farrand and Jones, 1996; Farrand et al., 2001).

**Table 1:** **Studies measuring forward and/or backward recall in verbal and/or visuospatial working memory**.

**Study**	**N**	**Age (Mean)**	**Gender (M/F)**	**Tasks**	**Behavioral measures**	**Main results**	**Order recall**	
							**F**	**B**
**STUDIES ON VERBAL WORKING MEMORY**								
Li and Lewandowsky, 1993	*N* = ranged from 12 to 30 (Study 1–4)	Undergraduate students	NA	List of words (Study 1, 2 and 4) or letters (Study 3) and distractor item(s) that involved adding 3 to a random number and saying the result aloud.	Correct responses (correct item reported in its initial serial position regardless of the number and the nature of any preceding responses).	Forward but not backward recall was disrupted by an intralist distraction task. This effect was greatest for the first and last items and tended to decrease for the last items.	✓	✓
Li and Lewandowsky, 1995	*N* = ranged from 10 to 15	Undergraduate students	NA	Consonants (Study 1 and 3) or words (Study 2) and distractor item(s) (Study 1 and 2).	Correct responses (correct item reported in its initial serial position regardless of the number and the nature of any preceding responses).	Differences between forward and backward recall are interpreted as supporting the existence of two different retrieval processes for forward and backward order.	✓	✓
Hulme et al., 1997	*N* = 28 (Study 4)	Undergraduate students	Study 4: 24 F, 4 M	List of short high-frequency words vs. short low-frequency words.	Proportion of correct recall.	Word frequency had an increasing effect across serial positions in forward but not in backward recall.	✓	✓
Bireta et al., 2010	*N* = 20	Undergraduate students	NA	Short vs. long words (Study 1); letters with no noise vs. irrelevant speech (Study 2); similar- vs. dissimilar-sounding letters (Study 3); words associated with quiet vs. concurrent AS (Study 4).	Proportion of correct responses.	Word length effect, irrelevant speech effect, acoustic confusion effect, and concurrent articulation effect were less strong in the backward than in the forward recall, or even disappeared.	✓	✓
Guérard et al., 2012	*N* = ranged from 20 to 40	Undergraduate students	NA	Phonologically similar vs. monosyllabic words (Study 1A and 1B) associated with quiet vs. irrelevant speech (Study 2); open vs. closed pool of words associated with quiet vs. concurrent AS (Study 3); short vs. long word condition (Study 4); monosyllabic vs. polysyllabic words (Study 5).	Proportion of correct responses.	A replication of the Bireta et al. study (2010) revealed word length effect, irrelevant speech effect, acoustic confusion effect and concurrent articulation effect in both forward and backward recall.	✓	✓
**STUDIES ON VISUOSPATIAL WORKING MEMORY**								
Vandierendonck et al., 2004	*N* = 25 (Study 1 and 2)	Undergraduate students	NA	Computerized CST with randomly selected block sequences of different lengths; Single vs. dual-task situations (AS, matrix-tapping, random-interval generation and fixed-interval generation) (Study 1 and 2).	Proportion of correct locations or not (Study 1 and 2).	The random interval generation task condition coincided with worse performance in backward than in forward order recall. Concurrently performing the matrix-tapping task impaired memory performance in both recall directions. Articulatory suppression did not affect memory performance on forward recall, but on backward recall, it impaired memory for longer sequences on backward recall. The standardized difference between forward and backward spatial span was 0.11 [−1.83, 2.04].	✓	✓
Cornoldi and Mammarella, 2008	*N* = 40	19.05–19.23 years (HSA-LSA group)	HSA: 2M, 18 F LSA: 1M, 19 F	CST using ascending vs. descending format and different sequence lengths.	Order scores in terms of mean percentages of correct responses.	Participants with low-spatial-abilities (LSA) showed specific impairment in the backward recall direction; no differences between forward and backward recall in the high-spatial-ability (HAS) group.	✓	✓
Garcia et al., 2014	*N* = 45	8–10 years	NLD:10 M, 5 F RD: 6 M, 9 F TD: 8M, 7 F	CST, Color task, Color-location binding task.	Percentages of correct responses.	The non-verbal learning disability (NLD) group performed worse than the typical development (TD) group in the CST, and particularly in backward recall. The standardized difference between forward and backward spatial span was −0.50 [−1.21, 0.24].	✓	✓
Higo et al., 2014	*N* ranged from 21 to 24 (Study 1 and 2)	22.7 years–22.9 years (Study 1 and 2)	Study 1: 11 M, 13 F, Study 2: 12 M, 9 F	CST associated with secondary tasks (control, serial AS or ST) (Study 2); item sequence length manipulation (Study 1).	Correct trials, position error, order errors (Study 1 and 2).	Spatial tapping (ST) interfered equally with both forward and backward recall, while serial articulatory suppression (AS) increased the number of order errors in backward recall, suggesting a stronger order representation for backward recall. The standardized difference between forward and backward spatial span was 0.40 [−0.01, 0.82].	✓	✓
**STUDIES ON VERBAL AND VISUOSPATIAL WORKING MEMORY**								
Jones et al., 1995	N ranged from 20 to 36 (Study 1, 2 and 3)	Undergraduate students	NA	Sequences of dots (Study 1); Verbal (consonant) vs. spatial memory (dots) task associated with verbal or spatial interference (spatial manual interference, AS, irrelevant speech, respectively for Study 2- 3-4) and steady state vs. changing state condition.	Serial order errors (Study 1, 2).	Similar position curves, more errors with increasing list length, and more errors when retention interval increased, in forward verbal and spatial span tasks.	✓	
Farrand and Jones, 1996	*N* = 18 (Study 1, 2, 3 and 4)	NA	NA	Spatial task (dot), Visual-Verbal Task (sequence of consonants) and Auditory-Verbal Task (sequence of recorded consonants) (Study 1); Only Visual-Verbal Task (sequence of consonants) (Study 2 and 3); Only Spatial task (dot) (Study 4).	Serial position curves, serial order errors, city block errors (Study 4).	Performance on forward and backward recall for verbal and spatial stimuli was similar when only the order of information had to be recalled (Study 1). When both item and order information had to be recalled, performance in backward recall was significantly worse than in forward recall, suggesting a similarity between verbal and spatial recall.	✓	✓
Wilde and Strauss, 2002	*N* = 44	*M* = 37.1 years (*SD* = 13.9)	26 M, 18 F	Digit span and Spatial span tests.	Digit span and Spatial span scores.	Better performance in forward than in backward recall in the Digit span but not in the Spatial span tasks.	✓	✓
Mammarella and Cornoldi, 2005	*N* ranged from 36 to 46 (Study 1 and 2)	3rd-, 4th-, and 5th-grade schoolchildren (Study 1); nd- and 5th- grade schoolchildren (Study 2)	Study 1: NLD: 12 M, 6 F and CG: 12 M, 6 F Study 2: NA (Study 1–2)	Digit span and CST test.	Correct responses (Study 1 and 2).	In two studies the NLD group showed significantly lower scores in the backward than in the forward CST. In the Digit span task, both groups performed worse in backward recall. In the first study, the standardized difference between forward and backward spatial span was −0.48 [−1.13, 0.24]. In the second study, the standardized difference was 0.17 [-0.61, 0.93] and 0.18 [−0.63, 0.98] for children in the second and in the fifth grade respectively.	✓	✓
Depoorter and Vandierendonck, 2009	*N* ranged from17 to 20 (Study 1, 2, 3, 4)	19.5–24.7 years	Study 1: 6 M, 11 F; Study 2: 5 M, 12 F; Study 3: 1 M, 19 F	Primary visuospatial task (visuospatial squares and lines task) associated with secondary visuospatial item or order task (Study 1); Primary verbal task (letters) associated with secondary verbal item or order task (Study 2); Primary visuospatial and verbal task associated with secondary verbal and visuospatial task (Study 3 and 4).	Correct responses (Study 1 and 2) and ratio measures (Study 2).	Worse performance in primary task recall when both the primary and the secondary task involved order, irrespective of whether input was verbal or visuospatial.	✓	
Vandierendonck, 2016	N ranged from 22 to 24 (Study 1 and 2)	18–22/32 years (Study 1 and 2)	Study 1: 2 M, 22 F Study 2: 20 M, 12 F	Verbal (one-syllable Dutch words) vs. visuospatial (dot task) as primary vs. secondary task with order vs. item recall stimuli (Study 1). Verbal serial primary task associated with secondary visuospatial task in different conditions (perfect embedded trials, primary task recall for all trials and embedded task recall) (Study 2).	Serial recall tasks: proportion of elements recalled in correct relative position (scoring between 0 and 1); item recall tasks were scored 0 or 1 (incorrect vs. correct).	Visuospatial, but not verbal serial recall was more impaired when the embedded task was an order recall task than when it was an item recall task (Exp. 1), suggesting that order is at least partly coded on a different, modality-independent level.	✓	

*Note: AS, articulatory suppression; DVN, dynamic visual noise; HSA, high spatial abilities; LSA, low spatial abilities; NA, not available; NLD, non-verbal learning disability; RD, reading disability; ST, spatial tapping; TD, typical development. Papers in this Table are arranged by year of publication*.

Several studies adopted the dual task paradigm when examining verbal tasks. In the literature, the most commonly used examples of dual tasks are *articulatory suppression*, and *irrelevant speech* (i.e., concurrent irrelevant sounds). These dual tasks effectively impair memory performance (see Baddeley and Hitch, 1974). In two separate studies, Bireta et al. (2010) and Guérard et al. (2012) explored these effects using similar methods and procedures, but came to different conclusions. In both studies, all the above-mentioned effects were confirmed on forward recall, but only Guérard et al. (2012) found the dual tasks had an impact on backward recall as well. Ritchie et al. (2015) conducted a meta-analysis of 16 experiments focusing on secondary tasks. Irrelevant speech was observed to have a weak effect on task performance. In contrast, articulatory suppression had very large effects, and seemed to disrupt recall for both first and late responses (i.e., primacy and recency), in forward and backward recall alike.

As for the visuospatial domain, studies tend to take one of two approaches: comparing extreme groups, or analyzing clinical populations (Table 1, visuospatial WM). For example, adults with high spatial abilities demonstrated very similar performance in the forward and backward versions of the CST (Wilde and Strauss, 2002), whereas participants with low spatial abilities demonstrated lower performances in backward recall (Cornoldi and Mammarella, 2008). This finding was also confirmed in children with non-verbal learning disability who had severe problems in the spatial domain (Cornoldi et al., 2003; Mammarella and Cornoldi, 2005; Garcia et al., 2014).

Other research directly compared the verbal and visuospatial domains (Table 1, verbal and visuospatial WM) by using the dual task paradigm. Research has shown that a serial secondary task (i.e., spatial tapping) interferes with recall of spatial information (Jones et al., 1995; Vandierendonck et al., 2004). Further, the presence of a verbal secondary task affects both verbal and visuospatial recall when the secondary task requires the manipulation of ordered information. However, when the secondary task requires the manipulation of unordered materials, visuospatial performance is not affected (Depoorter and Vandierendonck, 2009). This result was used by the researchers as evidence of the existence of a cross modal interference. Others argued that the effect seen in Depoorter and Vandierendonck's research was probably due to the specific manipulation used in the study (Logie et al., 2016). However, in further research a different manipulation was employed, and the results confirmed the existence of cross modal interference between verbal and spatial recall performance (Vandierendonck, 2016). This effect was also confirmed using visuospatial materials only (i.e., the CST), showing that both forward and backward recall were affected by the presence of a verbal secondary tasks (Higo et al., 2014).

### Neural correlates

Although, there is not a general consensus on a specific WM model, neuroscience studies can help to shed further light on the effect of order recall in verbal and visuospatial domains. In fact, event-related potentials (ERPs) and functional Magnetic Resonance Imaging (fMRI) have also been used to investigate neural correlates of forward and backward recall in the verbal and visuospatial domains (see Table 2).

**Table 2:** **Studies using fMRI or ERP measuring forward and/or backward recall in verbal and/or visuospatial working memory**.

**Study**	**N**	**Age (Mean)**	**Gender (M/F)**	**Tasks**	**Measures**		**Main results**	**Order recall**	
					**fMRI**	**ERP**		**F**	**B**
**STUDIES ON VERBAL WORKING MEMORY**									
Walhovd and Fjell, 2002	*N* = 71 divided in 5 age group	21.8–94.7 years	35 F, 36 M	Digit span task.		✓	Significant negative correlations were present between P3 latency and digit span scores. Moreover, a multiple regression analysis controlling for age yielded a significant relationship between P3 latency and both the digit total and forward span tasks.	✓	✓
Lefebvre et al., 2005	*N* = 20	22.2 years	11 F, 9 M	Digit span backwards task consisting of different set sizes was aurally presented, followed by a second set that either corresponded to the reverse order of the first set (correct condition) or had one digit in the sequence replaced by an incorrect digit (incorrect condition). The participants have to indicate whether the trial was correct or incorrect.		✓	Positive component (P2/P3) was elicited in the correct condition, whereas a comparatively robust and prolonged positive slow wave was elicited in the incorrect condition. Moreover, the second wave and the difference between its amplitude in the incorrect and correct conditions decreased as working memory load increased.		✓
Sun et al., 2005	*N* = 22 (Young adults: 11; Older adults: 11)	Young adults: 19–27 years; Older adults: 60–72 years	Young adults: 5 F, 5 M; Older adults: 5 F, 5M	Digit recall task.	✓		In young adults, the left occipital visual region and the left PFC were more activated during backward recall task than forward recall task, while the GFi was more activated in forward than backward direction. In older adults, more regions and especially the frontal cortex showed a greater activation in backward than forward digit recall, while the GFi was more activated during backward recall.	✓	✓
Majerus et al., 2006	*N* = 18	20–28 years	NA	Sequential presentation of four words with probe recognition for word identity or word order.	✓		An increase in lateral inferior parietal activation was observed when comparing order and item conditions. However, activation within the left IPS was observed in both the order and item tasks, while the left IPS was activated during order than item conditions.	✓	
Marchand et al., 2006	*N* = 20	22.2 years	11 F, 9 M	Digit span backwards task consisting of different set sizes was aurally presented, followed by a second set that either corresponded to the reverse order of the first set (correct condition) or had one digit in the sequence replaced by an incorrect digit (incorrect condition). The participants have to indicate whether the trial was correct or incorrect.		✓	A prolonged positive slow wave (PSW) peaking between 450 and 750 ms. was elicited to incorrect condition trials.		✓
Marshuetz et al., 2006	*N* = 12	20–27 years	6 F, 6 M	Five consonants were presented, followed by two probe letters from the memory set. The probe letters appeared either in the original order or were transposed and were separated by zero to three positions in the memory set. Participants had to indicate whether the items were in the correct order.	✓		The activation in the left parietal cortex showed a systematic decrease, with increasing inter-item probe distance.	✓	
Majerus et al., 2008	*N* = 22	HPB: 19.42 years; LPB: 19.62 years	HPB: 9 F, 2 M; LPB: 7 F, 4 M	Visual and sequential presentation of four words, followed by a maintenance phase, and a retrieval phase in which probe words were matching or not the target information.	✓		High proficiency bilinguals presented a greater activation in the lateral orbito-frontal and superior frontal gyri associated with order condition. High and low proficiency groups showed similar activation of fronto-parietal and fronto-temporal networks for order and item conditions. However, for order conditions, the high proficiency group showed a larger activation in the left orbito-frontal cortex during encoding, while the low proficiency group showed a greater activation in the bilateral superior frontal cortex during retrieval.	✓	
Rossi et al., 2013	*N* = 22	10 years	11 F, 11 M	Digit span task.	✓		The gray matter volume of the left AIC and the frontal and prefrontal regions were negatively correlated with backward memory span recall. On the other hand, the gray matter volume of the inferior parietal lobe was negatively correlated with the forward memory span. Moreover, smaller gray matter volumes in the left AIC region, the inferior and the superior frontal gyrus, were present in children who performed better on the backward span task.	✓	✓
Manan et al., 2014	*N* = 54	G1: 20–29 years; G2: 30–39 years; G3: 40–49 years; G4: 50–65 years	10 F, 44 M	Backward repeat task (BRT) and forward repeat task (FRT) where verbal stimuli consisted of a series of natural recorder speech words.	✓		Backward recall is associated with the activation of parietal and frontal regions compared to forward recall. Moreover, brain changes related to hemispheric laterality were detected in relation to age.	✓	✓
Yang et al., 2015	*N* = 72	7.13–16.84 years	32 F, 40 M	Digit span test.	✓		Backward digit span recall was associated with the activation of the right DLPFC, the FEF, the frontal operculum cortex, anterior insular cortex, and the dACC, while forward digit recall was associated with precuneus and lateral visual areas. Finally, the dACC region was positively related to backward span task but negatively related to forward span task.	✓	✓
**STUDIES ON VISUOSPATIAL WORKING MEMORY**									
Croizé et al., 2004	*N* = 8	28 years	4 F, 6 M	In the task, subjects decided whether two dots were symmetrical or not with respect to a central fixation cross. Dots were presented either simultaneously (“immediate condition”) or successively (“memorization” of the position of a flashed dot and a “delayed comparison” with a second displayed dot).	✓		The M4 activation observed by using magnetoencephalography (MEG) in the “memorization” condition was localized in the right premotor region, in direct spatial correspondence with the precise activation focus revealed by fMRI, along the premotor sulcus and at the intersection with the superior frontal sulcus.	✓	
Toepper et al., 2010	*N* = 19	26.1 years	10 F, 9M	Modified version of the CST. Participants were asked to reproduce each sequence by deciding which between two alternative response options.	✓		When compared to the baseline activity, the right hippocampus showed more activation during the CST condition. Moreover, increased activity within parietal, frontal and occipital areas was observed during the encoding stage.	✓	
Toepper et al., 2014	*N* = 36 (young: 18; old: 18)	young: 26.8 years, old: 71.7 years	young: 9 F, 9 M; old: 9 F, 9 M	Modified version of the CST where participants were asked to reproduce each sequence by pressing buttons in the correct order.	✓		When older individuals were compared to younger participants, lower right-dorsolateral prefrontal activation and lower functional connectivity between this area and the bilateral orbitofrontal cortex was observed. Moreover, the older high-performance group showed higher right dorsolateral and anterior prefrontal cortex activation and higher functional connectivity between these regions when compared to the older low-performance group.	✓	
**STUDIES ON VERBAL AND VISUOSPATIAL WORKING MEMORY**									
Nulsen et al., 2010	*N* = 24	23 years	18 F, 6 M	Digit and spatial span tasks. Participants had to decide whether a sequence of stimuli shown during the test phase matched or dis not match the initial studied items.		✓	A large effect on reversal recall was observed specifically on the digits task, with a smaller effect seen in the spatial span tasks. Moreover, the mean amplitude of the P3a component was larger to a statistically significant degree in the forward condition for the digit task, compared to the backward condition. The standardized difference between forward and backward spatial span was 0.26 [−0.15, 0.66].	✓	✓
Chein et al., 2011	*N* = 12	19–24 years	Study: 7 F, 5 M	For processing components: lexical decision and symmetry decision tasks; for storage components: letters and location tasks, in which subjects attempted to retain a sequence of 4 items for later recall.	✓		For both verbal and spatial versions of complex working memory span tasks, an increased activity in lateral prefrontal, anterior cingulate, and parietal cortices during the Encoding, Maintenance, and Coordination phase of task performance was seen. In addition, overlapping activity in the anterior prefrontal and medial temporal lobe regions was associated with both verbal and spatial recall from working memory.	✓	
Nagel et al., 2013	*N* = 67	10–16 years	32 F, 35 M	Block-design, verbal and spatial WM 2-back task that differed only by way of task instruction.	✓		While verbal WM was associated with significant left hemispheric lateralization in the frontal and parietal lobes, the spatial WM involved right hemisphere lateralization in the frontal and temporal areas. Moreover, increased adolescent age was associated with less activity in the default mode brain network during the verbal WM, and associated with greater activity in the task-positive posterior parietal cortex during spatial WM task.	✓	

*Note: ACC, anterior cingulate cortex; AIC, anterior insular cortex; CWMS, complex working memory span; dACC, dorsal anterior cingulate gyrus; DLPFC, dorsolateral prefrontal cortex; FEF, frontal eye fields; GFi, inferior frontal gyrus; HPB, High proficiency bilinguals; IPS, left intraparietal sulcus; LPB, low proficiency bilinguals; NA, not available; PFC, prefrontal cortex; PPC, posterior parietal cortex. Papers in this Table are arranged by year of publication*.

ERPs were used in the backward DST under two conditions; digits were aurally presented and were followed by a second set that either corresponded to the reverse order (correct condition), or by a second set in which an incorrect digit was included in the list (incorrect condition) (Lefebvre et al., 2005; Marchand et al., 2006). The findings showed a positive P2 and P3 in the correct condition, and conversely showed a prolonged positive slow wave for the incorrect condition; this suggests that the two conditions are associated with different patterns of activation. Another study compared forward and backward recall, showing the presence of high negative correlations between P3 latency and the DST (Walhovd and Fjell, 2002). Finally, research has shown that the amplitudes of the P3a and P3b ERPs are reduced during backward recall in verbal but not in visuospatial tasks (Nulsen et al., 2010). This finding, suggesting a different pattern of activation between verbal and spatial backward spans, seems to indicate a reduction of attentional resources in the verbal backward span (see Nulsen et al., 2010).

As for fMRI studies, in the verbal domain, two studies compared the recognition of ordered and item information. In the ordered condition, results show a greater bilateral activation in the intraparietal sulcus (IPS) and in the premotor frontal areas (Henson et al., 2000; Marshuetz et al., 2000), supporting the idea that ordered material requires more attentional resources. However, a study by Majerus et al. (2006) failed to find a consistent differential activation in the left IPS, indicating that the difference between order and item condition is related to a specific network that links the left and right IPS with the right dorsal premotor cortex and the superior cerebellum. Interestingly, bilingual individuals with a high level of proficiency in both languages demonstrated greater activation in the lateral orbito-frontal region and in the superior frontal gyri associated with the updating of ordered information (Majerus et al., 2008), confirming the presence of different patterns of activation for order and item encoding.

Studies comparing different patterns of activation in forward and backward recall are mainly focused on verbal material. Research has shown the involvement of different neural correlates in forward and backward recall of digits (Manan et al., 2014). Another study showed that backward digit recall was associated with a higher activation of the left occipital visual region and the left prefrontal cortex (PFC) in young adults (Sun et al., 2005), supporting the idea of the involvement of visuospatial processing during backward verbal tasks (e.g., Larrabee and Kane, 1986; Hoshi et al., 2000). Moreover, young adults showed a greater activation in the inferior frontal gyrus in both forward and backward recall. The activation was associated with a limited overlap, providing evidence in favor of a distinction between forward and backward recall activation patterns (Sun et al., 2005). Furthermore, the central executive seems to be highly taxed during backward digit recall (Carlesimo et al., 1994). These results are in line with previous findings suggesting that the backward DST was associated with the activation of regions that are also involved in tasks requiring high cognitive control. Such activated regions include the right dorsolateral PFC, the frontal eye field, the frontal operculum cortex, the anterior insular cortex and the dorsal anterior cingulate cortex (dACC) (Yang et al., 2015). Intriguingly, activation of the dACC region was positively related to the backward span task but negatively related to the forward one (Yang et al., 2015). Finally, results with child subjects revealed the presence of distinct negative correlations between the forward/backward DST and the gray matter volume of some brain areas, such as the left AIC region, the inferior frontal gyrus and the superior frontal gyrus (Rossi et al., 2013).

While research on verbal material is plentiful in the literature, fMRI studies on the visuospatial domain are mainly focused on the forward span. In a study, when participants were asked to decide whether two dots were symmetrical or not, the results revealed that in the memorization condition, where participants had to judge whether the symmetry of the second dot's position related to the memorized position of the first dot, the right premotor region was activated (Croizé et al., 2004). Two other studies involved a modified version of the CST, and showed the involvement of the hippocampus in the encoding of spatial locations (Toepper et al., 2010). In addition, age effects were observed in the right-dorsolateral prefrontal cortex, which was found to be less activated in the older group compared to the younger one (Toepper et al., 2014).

Finally, in two studies comparing verbal and visuospatial domains the results seemed to favor a distinction between the two domains (Chein et al., 2011; Nagel et al., 2013). For example, Nagel et al. (2013) considered the difference between the verbal and visuospatial domains from a developmental point of view, suggesting that increased adolescent age was associated with less activity in the default mode brain network (i.e., a brain network more commonly active at rest and deactivated during task) during a verbal WM task. In contrast, increased adolescent age was associated with greater activity in the posterior parietal cortex during a spatial WM task.

### Implications for the working memory models

The presence of different results for order recall in verbal and visuospatial domains is considered as evidence in support of several existing theoretical models of WM. Baddeley's WM model postulates the existence of two domain-specific subsystems involved in the storage of verbal and visuospatial information: the *phonological loop* and the *visuospatial sketchpad*, respectively. These two components are linked with the *central executive system* that integrates and manipulates information (e.g., Baddeley, 1986). In this model, the *phonological loop* explains several phenomena affecting serial recall in verbal STM, such as the influence of word length, articulatory suppression, phonological similarity and item similarity. The decline in performance in the backward span is also interpreted in relation to the central executive's taxed resources (Baddeley, 1986). However, a limit of this model is that it fails to explain the results observed in visuospatial tasks and lacks a clear distinction between recalling sequential ordered information, and recalling unordered information.

Alternative models of WM propose a modality-independent view, with no distinction between verbal and visuospatial input. This approach is supported by the similar serial position curves detected in the verbal and visuospatial domains, and the shared memory resources for maintaining information in a given order (Engle, 1996; Cowan, 1999, 2005; Oberauer, 2009). It has also been suggested that the difference between verbal and visuospatial span tasks in forward and backward directions is associated with dissimilar retrieval demands: while participants use blocks to give their answers in the CST, in the DST the digits are not presented during the retrieval phase. Thus, verbal tasks would seem to require the recall of both items and order information, while visuospatial tasks would only require the latter (Farrand and Jones, 1996). Similarities between serial order and position effects in the verbal and spatial domains can be explained by assuming that order is treated similarly across different domains (Smyth, 1996). A comparable view is based on the assumption that there is a modality-independent process for serial order retention, and a domain-specific process for item retention (Depoorter and Vandierendonck, 2009).

Another hypothesis postulates that verbal and visuospatial forward serial recall measures the “passive” STM component, while backward recall involves executive control resources (Carlesimo et al., 1994; Hester et al., 2004). Developmental studies, combined with research in which clinical samples were considered, have helped to clarify this hypothesis. For example, a greater involvement of executive control in backward serial recall has been demonstrated in typically-developing children (Alloway et al., 2009), and in children with ADHD or learning disabilities (Cornoldi et al., 2013a,b; Giofrè et al., 2016), but not in adults (Rosen and Engle, 1997).

Concerning the visuospatial domain, a model has been proposed (Logie, 1995; Darling et al., 2007) that distinguishes between the *visual cache*, linked with the temporary storage of static visual information, and the *inner scribe*, involved in the dynamic processing of sequences of movement. According to this model, the maintenance of sequential information is crucial in spatial processes. This is in contrast with other models which are based on the assumption that visuospatial processes tend to lose sequential information in favor of simultaneously presented information (Paivio, 1971).

Finally, a model distinguishing between a visual component and two spatial subcomponents involving spatial-sequential and spatial-simultaneous processes has been proposed (Lecerf and De Ribaupierre, 2005; Mammarella et al., 2008, 2013). This is supported by findings in different groups of children with developmental disorders (Mammarella et al., 2003, 2006; Lanfranchi et al., 2015), and in healthy adults (Mammarella et al., 2013). In this view, Mammarella and Cornoldi (2005) suggested that differences in forward and backward spatial recall are due not only to the involvement of the executive control, but also to involvement of spatial-sequential and spatial-simultaneous processing. Research has also shown that backward recall in the CST requires less executive control and more spatial processing, supporting the idea that backward recall involves a modality-independent order coding system (Higo et al., 2014). This hypothesis is supported by evidence suggesting that the backward CST involves both visuospatial processing and executive control (Vandierendonck et al., 2004; Vandierendonck, 2016).

We decided to investigate this result further by analyzing papers including both versions of the CST. Nine papers included in this review deal with both versions of the spatial span. Among these studies, two did not report means or effect sizes, making it impossible to calculate effect sizes (Farrand and Jones, 1996; Farrand et al., 2001); one reported data from a clinical sample (Wilde and Strauss, 2002), and one compared participants with high vs. low spatial abilities (Cornoldi and Mammarella, 2008). However, five studies reported descriptive statistics (i.e., Vandierendonck et al., 2004; Mammarella and Cornoldi, 2005; Nulsen et al., 2010; Garcia et al., 2014; Higo et al., 2014) and seven effect sizes were extracted from these studies (see also Tables 1, 2). When considering these effects together, and assuming random effects, the overall effect is *d*_*unb*_ = 0.039 [-0.20, 0.28]. This finding seems to indicate the difference between forward and backward spatial span is very small and not statistically significant.

## Conclusions

In the present paper, we reviewed findings on forward and backward recall in the verbal and visuospatial domains, considering the contribution of experimental and neuroscience studies.

The evidence from the cognitive studies is quite clear. Regarding the verbal domain, the verbal recall task is often characterized by a clear difference between the forward and the backward version of the span, with lower performance in the latter. In the visuospatial domain—at least when typically developing children or healthy adults are considered—it is more difficult to detect differences between recall of the forward and backward versions of the task.

Overall, experimental studies do not provide a clear support for any theoretical model described above. Advances in technical and quantitative methods of neuroscience over the past years have aided and propelled analysis in various fields of psychology. Neuroscientific studies cited in this review have indicated that verbal recall, in the backward order in particular, seems to require greater cognitive resources (Manan et al., 2014). In addition, different brain areas are activated in verbal and visuospatial tasks (Sun et al., 2005; Yang et al., 2015). These findings support modality independent models of WM, and in fact verbal performance and visuospatial performance is always clearly distinguishable.

Unfortunately, to-date no study has compared forward and backward recall in verbal and visuospatial domains in relation to neural correlates. A promising future line of research would involve studies that examine the simultaneous storage of information derived from different modalities. In fact, future efforts should directly compare the neural correlates of forward and backward recall in verbal and visuospatial domains, and do so within a single study. Furthermore, other techniques should be used in order to collect further evidence. Potentially, future studies could employ other psycho-physiological measures such as eye movements, or neuroimaging techniques such as transcranial magnetic stimulation or magneto-encephalography. These kinds of additional analytical measures could allow researchers to reach clearer results. Moreover, methodologies and the types of tasks used in future studies should be consistent and comparable.

Ultimately, only few developmental studies have been carried out to-date; therefore, how the serial recall of verbal and spatial information develops is not yet completely clear. A deeper understanding of such changes could in turn help in improving our understanding of currently existing theoretical models.

Despite some shortcomings, the findings collectively gathered in this review are both comprehensive and beneficial to those currently researching in this field. The take home messages from these reviews are as follows: (1) verbal and spatial WM modalities seem to be distinct; (2) there is overwhelming evidence for a distinction between the forward and backward digit span; and (3) there is no clear evidence for a distinction between forward and backward spatial span.

## Author contributions

ED, ICM, and DG contributed to the study concept and design. ED, ICM, and DG wrote the paper. All authors approved the final version of the manuscript for submission.

### Conflict of interest statement

The authors declare that the research was conducted in the absence of any commercial or financial relationships that could be construed as a potential conflict of interest.
